# Improvement of Transmembrane Transport Mechanism Study of Imperatorin on P-Glycoprotein-Mediated Drug Transport

**DOI:** 10.3390/molecules21121606

**Published:** 2016-11-24

**Authors:** Zheng-Gen Liao, Tao Tang, Xue-Jing Guan, Wei Dong, Jing Zhang, Guo-Wei Zhao, Ming Yang, Xin-Li Liang

**Affiliations:** Key Laboratory of Modern Preparation of TCM, Jiangxi University of Traditional Chinese Medicine, Nanchang 330004, China; lyzlyg@163.com (Z.-G.L.); tangtao12306@sina.com (T.T.); guanxj52611905@sina.com (X.-J.G.); sober96@foxmail.com (W.D.); evens_zhang@163.com (J.Z.); weiweihaoyunqi@163.com (G.-W.Z.); doscat@163.com (M.Y.)

**Keywords:** imperatorin, P-glycoprotein (P-gp), transmembrane transport, mechanism

## Abstract

P-glycoprotein (P-gp) affects the transport of many drugs; including puerarin and vincristine. Our previous study demonstrated that imperatorin increased the intestinal absorption of puerarin and vincristine by inhibiting P-gp-mediated drug efflux. However; the underlying mechanism was not known. The present study investigated the mechanism by which imperatorin promotes P-gp-mediated drug transport. We used molecular docking to predict the binding force between imperatorin and P-gp and the effect of imperatorin on P-gp activity. P-gp efflux activity and P-gp ATPase activity were measured using a rhodamine 123 (Rh-123) accumulation assay and a Pgp-Glo™ assay; respectively. The fluorescent probe 1,6-diphenyl-1,3,5-hexatriene (DPH) was used to assess cellular membrane fluidity in MDCK-MDR1 cells. Western blotting was used to analyze the effect of imperatorin on P-gp expression; and P-gp mRNA levels were assessed by qRT-PCR. Molecular docking results demonstrated that the binding force between imperatorin and P-gp was much weaker than the force between P-gp and verapamil (a P-gp substrate). Imperatorin activated P-gp ATPase activity; which had a role in the inhibition of P-gp activity. Imperatorin promoted Rh-123 accumulation in MDCK-MDR1 cells and decreased cellular membrane fluidity. Western blotting demonstrated that imperatorin inhibited P-gp expression; and qRT-PCR revealed that imperatorin down-regulated P-gp (MDR1) gene expression. Imperatorin decreased P-gp-mediated drug efflux by inhibiting P-gp activity and the expression of P-gp mRNA and protein. Our results suggest that imperatorin could down-regulate P-gp expression to overcome multidrug resistance in tumors.

## 1. Introduction

Multidrug resistance (MDR) is a major cause of chemotherapy failure, and P-glycoprotein (P-gp) up-regulation is a classical MDR mechanism in cancer. P-gp is a 170–180-kDa protein that is encoded by the human MDR1 gene [1,2,3]. P-gp is in the ATP-binding cassette transporter superfamily, and it expels exogenous substances, including active drug components, such as important chemotherapeutic agents out of cells, using the energy released from enzymatic ATP hydrolysis. Therefore, P-gp reduces drug concentrations in cells [4,5,6]. P-gp is widely expressed in the epithelial cells of normal tissues that are involved in drug clearance, including the intestine, liver, and kidney, and it provides a barrier to sites such as the intestine and brain. P-gp is also highly expressed in cancer cells [7,8]. P-gp is selectively distributed in the intestinal mucosa, which is a key site of drug absorption, and the efflux function of P-gp establishes an important barrier against intestinal drug transport. Many orally administered drugs and active components, including drugs with strong pharmacological activity, e.g., doxorubicin, taxanes, vincristine, and peoniflorin, have poor bioavailability as a result of P-gp efflux, which makes oral preparation development difficult [9,10,11]. The study of these substances and tactics for overcoming or regulating P-gp efflux is important for increasing oral bioactivity and aiding in novel drug development [12,13]. The use of herbal medicines has dramatically increased in the past several years worldwide, and more than 80% of the population in developing countries relies on herbal and other traditional medicines for primary healthcare [14]. More people are using Traditional Chinese Medicine (TCM) to prevent and treat diseases in China. TCM studies and clinical experience demonstrate that many herbs, active ingredients of TCM, and TCM preparations exhibit positive effects in changing intestinal absorption and transmembrane transport. Researchers found that some Chinese herbs, such as Borneolum syntheticum, *Glycyrrhiza uralensis* Fisch, and *Angelica sinensis* (Oliv.) Diels [15] increase the absorption and retention of other drugs. Some Chinese herbal medicinal ingredients, such as dauricine, daurisoline [9,16], quercetin, and genistein [11,17], can inhibit P-gp efflux, but studies have not investigated the mechanism of P-gp efflux inhibition.

*Angelica dahurica* is used as a common ingredient in prescriptions such as *yuanhuzhitong-pian*, *duliang pill*, and *tongqiaobiyan-pian*, in the Chinese pharmacopeia. *Angelica dahurica* exhibits a protective effect against different types of pain [18]. Modern pharmaceutical studies have identified imperatorin as a major bioactive constituent that exhibits analgesic [19], anti-bacterial [20], anti-inflammatory [21], anti-tumor [22], vessel dilating [23], and CYP450 inhibitory effects [24]. Our previous studies examined the effect of *Angelica dahurica* on the intestinal absorption of P-gp-mediated drugs using in vivo and in vitro absorption models. Our results demonstrated that *Angelica dahurica* inhibited P-gp-mediated drug efflux. Our studies also indicated that imperatorin increased the instestinal absorption of puerartrin and vincristin sulfate, respectively, which is substrates of P-gp-mediated efflux [25,26]. The mechanism by which imperatorin inhibits the efflux of P-gp substrates is not known. Therefore, this study investigated the mechanism by which imperatorin inhibits P-gp-mediated drug efflux.

The MDCK-MDR1 cell line is derived from Madin-Darby canine kidney cells transfected with the human *MDR1* gene. The characteristics of these cells include high P-gp expression and rapid differentiation. MDCK-MDR1 cells are used as an alternative to the Caco-2 model for the high-throughput screening of permeability and drug-drug interactions in drug discovery [27]. This study used MDCK-MDR1 cells to investigate the mechanism by which imperatorin inhibits P-gp-mediated drug efflux. This study established the MDCK-MDR1 cell model to investigate the effect of imperatorin on the intracellular accumulation of Rh-123 in MDCK-MDR1 cells. The effect of imperatorin on P-gp ATPase activity was also investigated. Western blotting was performed to analyze the effect of imperatorin on P-gp expression and further confirmed imperatorin as a P-gp inhibitor. The mRNA levels of P-gp were assessed using qRT-PCR. The fluorescent probe DPH was used to detect the cellular membrane fluidity of MDCK-MDR1 cells. 

The present study was undertaken to explain the mechanism BY WHICH imperatorin improved the transport of the drugs in which transport is affected by the excretion of P-gp. The research could provide a feasible method for the research and development of potential P-gp inhibitors and could offer a foundation for imperatorin to be used as a P-gp mediated MDR reversal agent in the future.

## 2. Results

### 2.1. Western Blotting to Detect P-gp Expression in MDCK and MDCK-MDR1 Cells

**Figure 1:**
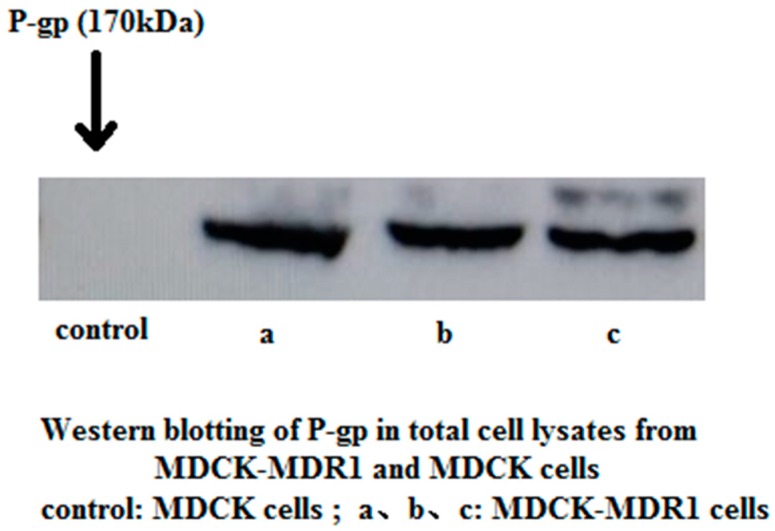
Comparison of the expression of P-gp in MDCK-MDR1 and MDCK native cells.

Total cellular protein was extracted from MDCK and MDCK-MDR1 cells, and P-gp expression was detected using western blotting. An obvious protein band of 170 kDa was observed in MDCK-MDR1 cell extracts, but no band was present at the corresponding position in MDCK extracts (Figure 1).

### 2.2. Molecular Docking Experiment to Detect the Association of Imperatorin and P-gp

**Figure 2:**
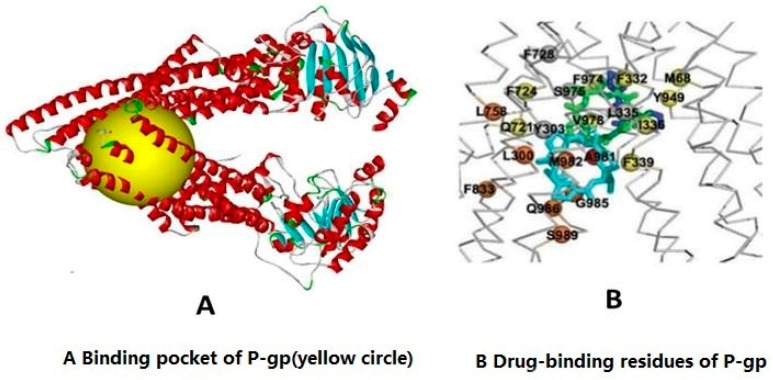
Binding pocket and drug-binding residues of P-gp. (**A**) Binding pocket of P-gp (yellow circle); (**B**) Drug-binding residues of P-gp. Yellow circle: binding pocket; Red chain: NBD1; Blue chain: NBD2; The number corresponding to the color is the P-gp protein drug binding site of amino acid residues in the intracellular nucleotide-binding domain.

Libdock (Discovery Studio version 4.0, Accelrys, Scottsdale, AZ, USA) is commercial software that can assess the docking of ligands into the active sites of proteins. The hotspot map for the protein active site, which includes polar and non-polar groups, is calculated. This hotspot map is then used to align the ligands that form favorable interactions. Libdock performs energy minimizations for all ligand positions and ranks the ligands based on each ligand’s score [28,29,30]. Figure 2 shows the binding pocket and drug-binding residues of P-gp. Table 1 shows the docking analysis. The results revealed an interaction between imperatorin and P-gp. Numerous interactions were predicted between imperatonin and residues in the binding pocket. Verapamil has a higher LibDock Score than imperatorin, which speculated that the strength of its binding to P-gp is greater than the binding between imperatorin and P-gp. Therefore, the mechanism by which imperatorin inhibits P-gp-mediated drug efflux may not be related to competitive binding with P-gp.

### 2.3. Effect of Imperatorin on P-gp Atpase Activity 

**Figure 3:**
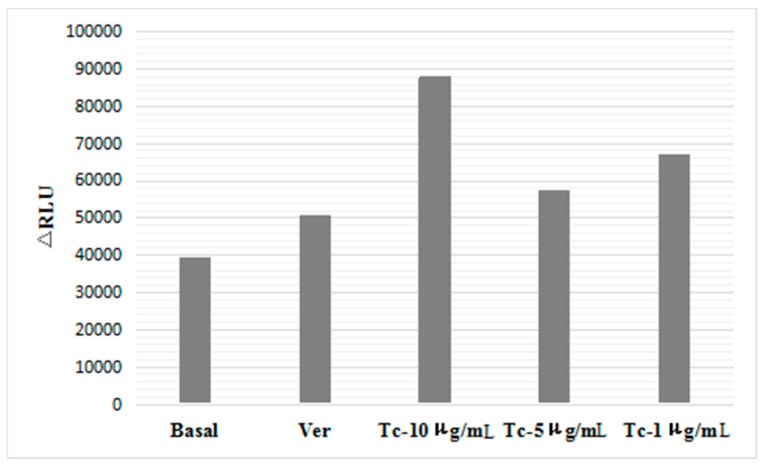
Fluorescence Intensity of control groups (*n* = 4).

Figure 3 shows the results. The ΔRLU_TC_ values of different imperatorin concentrations were higher than ΔRLU_basal_, indicating that imperatorin activated P-gp ATPase activity. This activation likely plays a role in the inhibition of P-gp-mediated drug efflux. Ver is a substrate of P-gp that also activates its ATPase activity. We concluded that imperatorin enhanced ATPase activity in a similar manner as Ver.

### 2.4. Effect of Imperatorin on Membrane Fluidity 

Cholesterol reduces membrane fluidity, and benzyl alcohol increases membrane fluidity. Our data demonstrated that the fluorescence anisotropy (r) of DPH in cholesterol-treated cells increased at least 20%, whereas the fluorescence anisotropy (r) of DPH in benzyl alcohol-treated cells reduced at least 6%. Different imperatorin concentrations (1 µg/mL, 5 µg/mL, and 10 µg/mL) increased the anisotropy values of cell membranes, which indicates that imperatorin reduced membrane fluidity (Table 2).

### 2.5. Inhibition of Rh-123 Efflux by Imperatorin

**Figure 4:**
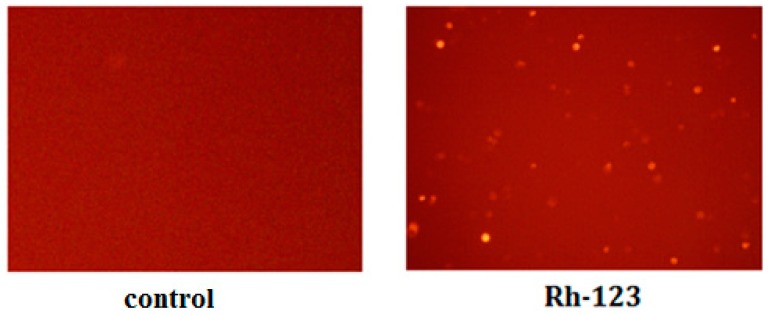
Fluorescent density of cells at the present of Rh-123 or not.

Rh-123 is a well-established P-gp substrate. P-gp induces Rh-123 efflux from cells and reduces Rh-123 accumulation inside cells. The decreased accumulation of Rh-123 indicates higher P-gp activity, whereas greater accumulation of Rh-123 indicates lower P-gp activity. MDCK-MDR1 cells were exposed to 5 µM Rh-123 and various concentrations of verapamil or imperatorin, and Rh-123 retention was determined by observation with an inverted fluorescent microscope (Figure 4). The treatment of MDCK-MDR1 cells with imperatorin significantly and dose-dependently increased Rh-123 accumulation (Table 3). 

### 2.6. Effect of Imperatorin on P-gp Protein Expression

**Figure 5:**
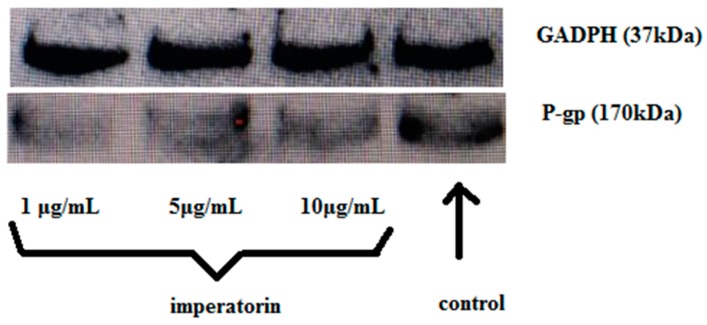
Effect of imperatorin on P-gp expression level.

P-gp expression levels were investigated by western blotting and bands were photographed. Figure 5 show that imperatorin decreased P-gp expression compared to the control group.

### 2.7. Effect of Imperatorin on P-gp mRNA Levels

**Figure 6:**
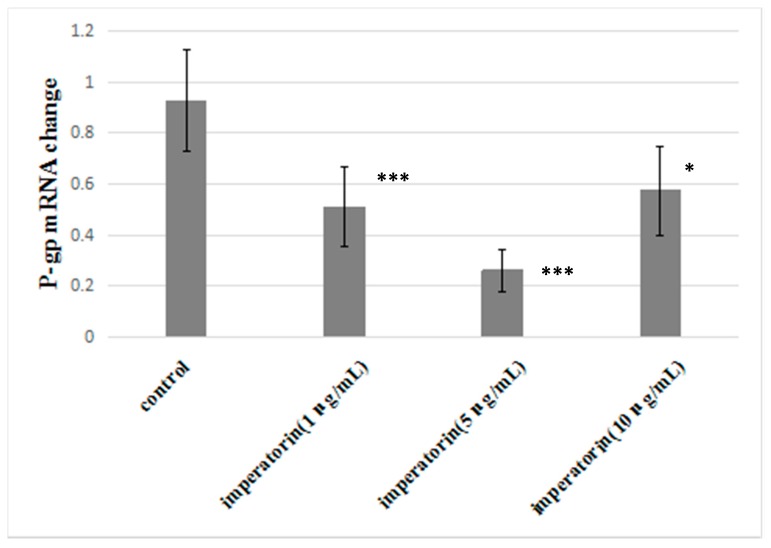
Effect of imperatorin on P-gp mRNA expression in MDCK-MDR1. Statistical significance is denoted * *p* < 0.05, *** *p* < 0.001 compared with control group.

Figure 6 shows the statistical analysis of P-gp mRNA levels. Imperatorin significantly reduced P-gp mRNA expression compared to the control group. However, imperatorin did not exhibit dose dependence in reducing P-gp mRNA expression.

## 3. Discussion

Changes in protein and mRNA expression levels can affect drug absorption. The physical state of cell membranes affects the function of membranes, including cell membrane permeability and enzyme activity in cell membranes [31]. High levels of P-gp expression and rapid differentiation are characteristics of MDCK-MDR1 cells. The present study investigated the effects of imperatorin on P-gp ATPase activity, membrane fluidity, Rh-123 accumulation, and P-gp mRNA and protein expression levels. Our results demonstrated that imperatorin increased P-gp ATPase activity, reduced membrane fluidity, increased Rh-123 accumulation, and reduced P-gp mRNA and protein levels in MDCK-MDR1 cells. The mechanism by which imperatorin inhibits P-gp-mediated drug transport may be related to P-gp ATPase activity induction, reduced membrane fluidity, and the reduced function or expression of P-gp. Our results demonstrate that the inhibition of P-gp-mediated drug transport by imperatorin involves multiple targets.

P-gp is an ATP-dependent transport protein that uses the energy derived from ATP hydrolysis to enable the efflux of hydrophobic and lipophilic drugs from cells. Verapamil is a substrate of P-gp that induces ATPase activity and is expelled from cells by P-gp. The results of our molecular docking experiments suggest that the inhibition of P-gp-mediated drug transport by imperatorin may not be related to competitive binding with P-gp but to the activation of the P-gp ATPase, which plays a role in the inhibition of P-gp function. 

Changes in membrane fluidity also influenced the activity of P-gp. Increased membrane fluidity can increase ATPase activity, and this P-gp-dependent enhanced ATPase activity can result in decreased drug efflux [32]. This study found that imperatorin significantly reduced MDCK-MDR1 cell membrane fluidity. The MDR reversal mechanism of imperatorin may be related to the reduced membrane fluidity of MDCK-MDR1 cells. A reduction in membrane fluidity is likely to affect the efflux function of P-gp.

Rh-123 is a liposoluble fluorescent substrate of P-gp. P-gp induces the efflux of Rh-123 out of cells. Rh-123 accumulation in cells is decreased by increased P-gp activity, whereas decreased P-gp activity results in greater Rh-123 accumulation. This method is widely used for the study of P-gp function [33]. Initially, 30 min, 1 h, and 2 h of incubation time were investigated and found that the incubation times have no effect on the results of the experiment. Therefore, the shortest time was selected that will allow to evaluate the potential immediate effects of the tested compounds on P-gp activity as a result of a direct activation of the pump. We selected the P-gp inhibitor verapamil as a positive control drug. Our data demonstrated that verapamil increased Rh-123 accumulation. 

In general, protein activity, protein expression, and gene expression are often closely related. In addition to directly inhibiting P-gp function, many P-gp inhibitors strongly decrease the mRNA and protein levels of P-gp [34]. Therefore, the effect of imperatorin on P-gp protein and mRNA expression was measured. Western blotting directly confirmed that imperatorin strongly down-regulated P-gp protein expression in MDCK-MDR1 cells, and qRT-PCR indicated that imperatorin also down-regulated P-gp mRNA expression.

Overall, our results suggest that imperatorin is a substrate and strong inhibitor of P-gp. The mechanism by which imperatorin inhibits P-gp-mediated drug transport may be related to the down-regulation of P-gp mRNA and protein expression in addition to the inhibition of efflux activity. 

## 4. Materials and Methods 

### 4.1. Chemicals and Drugs

Imperatorin was purchased from the National Institutes for Food and Drug Control (Beijing, China). Verapamil and Rh-123 were purchased form Sigma-Aldrich (St. Louis, MO, USA). Fetal bovine serum (FBS) and Dulbecco’s modified Eagle’s medium (DMEM) were purchased from Hyclone (Thermo Fisher Scientific, Beijing, China). Non-essential amino acids (NEAA) and verapamil hydrochloride were purchased from Sigma Chemical Co. Ltd. (Sigma, Germany). Trypsin-EDTA solution (0.25% (*w*/*w*) trypsin and 1 mM EDTA) was obtained from Gibco Laboratories (Life Technologies Inc., Beijing, China). Penicillin and streptomycin solutions (10,000 U/mL penicillin and 10,000 μg/mL streptomycin), MTT, and Hank’s Balanced Salts Solution (HBSS) were purchased from Beijing Solarbio Science & Technology Co. Ltd. (Beijing, China). Trizol RNA extraction kit was purchased from Ambion (Thermo Fisher Scientific, Beijing, China). The reverse transcription kit was purchased from Promega (Madison, WI, USA). Power SYBR Green PCR Mix was purchased from Life Technologies, Inc. PCR primers were synthesized by Shanghai Sangon Biotech Co. Ltd. (Shanghai, China). A mouse monoclonal anti-P-gp (MDR) antibody was obtained from Sigma-Aldrich, Inc. The GAPDH mouse monoclonal antibody was purchased from Shanghai Bioleaf Biotech Co. Ltd. (Shanghai, China). Horseradish peroxidase (HRP)-conjugated goat anti-mouse IgG, RIPA lysis buffer, protease inhibitors, phosphatase inhibitors, the BCA Protein Assay Reagent Kit, and the ECL Kit were purchased from Beijing Com Win Biotech Co. Ltd. (Beijing, China). P-gp-Glo™ assay systems were obtained from Promega. Soy lecithin and cholesterol were purchased from Tianjin Bodi Chemical Co. Ltd. (Tianjin, China). 1,6-Diphenyl-1,3,5-hexatriene (DPH) was purchased from Sigma-Aldrich, Inc.

### 4.2. Establishment and Validation of the MDCK-MDR1 Cell Model

#### 4.2.1 MDCK Cell Culture

MDCK cells were purchased from the American Type Culture Collection (Rockefeller, MD, USA). The cells were grown in an atmosphere of 5% CO_2_ at 37 °C. Cells were cultured in medium containing DMEM (high d-glucose 4.5 g/L), 10% fetal bovine serum, 1% non-essential amino acids, 1% l-glutamine, and a penicillin-streptomycin double antibiotic solution. Cell medium was changed daily. Cells were digested with 0.25% EDTA-trypsin and passaged at approximately 80%–90% confluence.

#### 4.2.2 MDCK-MDR1 Cell Culture

MDCK-MDR1 cells were purchased from the Shanghai Zhongya Biological Institute (CinoAsia co., Ltd.). The culture conditions were the same as for MDCK cells.

#### 4.2.3 Western Blotting to Detect P-gp Expression in MDCK and MDCK-MDR1 Cells

Cells were cultured on 12-well plates for at least 4–5 days, and the monolayers were washed 3 times with PBS. Total protein was extracted and measured. Protein (120 μL) was combined with 30 μL loading buffer (4×) in a 70 °C water bath for 10 min to completely denature the proteins. Electrophoresis was performed in 10% SDS-PAGE gels, and proteins were semi-dry transferred to membranes. Membranes were blocked in a blocking solution (5% powdered non-fat milk) for 4 h at room temperature and incubated overnight at 4 °C with an anti-P-gp antibody. NADPH (Santa Cruz Biotechnology, Shanghai, China) was used as a loading control. Membranes were incubated with goat anti-mouse IgG (1:5000 dilutions) for 2 h at room temperature. An ECL chromogenic solution was added and incubated for 4 min in the dark. The bands were photographed, and intensity was quantified using densitometry in Image Lab 3.0 (Beta 3, Bio-Rad, Irvine, CA, USA).

### 4.3. Molecular Docking

The molecular structures for the ligands imperatorin and verapamil were found in NCBI [35], and the SDF file format for molecular docking was downloaded. The X-ray crystal structure (4.40 Å resolution) of P-gp (3G60.PDB) was retrieved from the RCSB Protein Data Bank [36]. Its structure consists of two homodimeric chains, A and B. Discovery Studio 4.0 (DS) was used to perform the docking of the compounds into the active sites of P-gp. The protein was prepared by removing water and other hetero-atoms, adding hydrogen, and then performing protonation, ionization, and energy minimization. The CHARMmforce field was applied for geometry optimization. Met68, leu300, tyr303, phe332, leu335, ile336, phe339, gln721, phe724, phe728, leu758, phe833, tyr949, phe974, ser975, val978, ala981, met982, gly985, gln986, and ser989 were the amino acid residues of P-gp that were defined as active sites in these studies [37]. The detailed binding modes of ligands with P-gp were illustrated by docking all of the prepared ligands at the defined active site using Lab Dock (Wiebetech, Vancouver, WA, USA), and the Libdock scores were recorded for analysis [38,39].

### 4.4. P-gp ATPase Activity Assay

P-gp ATPase activity was measured using the luminescent Pgp-Glo™ Assay System (Promega, Madison, WI, USA) [40]. P-gp is an integral plasma membrane protein that functions as an ATP-dependent drug efflux pump and plays an important role in multi-drug resistance and certain adverse drug-drug interactions. Compounds that interact with P-gp may be stimulators or inhibitors of its ATPase activity.

The P-gp-Glo™ Assay detects the effects of compounds on recombinant human P-gp in a cell membrane fraction. The assay relies on the ATP dependence of the light-generating reaction of firefly luciferase. ATP is incubated with P-gp, and then the P-gp ATPase reaction is stopped. The remaining unmetabolized ATP is detected as a luciferase-generated luminescent signal. A P-gp-dependent decrease in luminescence reflects ATP consumption by P-gp. Therefore, a greater decrease in the signal indicates higher P-gp activity. Samples containing compounds that stimulate the P-gp ATPase exhibit significantly lower signals compared to untreated samples.

The P-gp-Glo™ Assay System included recombinant human P-gp membranes, ATP detection substrate, ATP detection buffer, Pgp-Glo™ assay buffer, MgATP, verapamil, and Na_3_VO_4_. Buffers and solutions were prepared and experimental studies were performed according to manufacturer guidelines. 

The D-value of the average luminescence intensity for the Na_3_VO_4_ group (RLU_Na3VO4_) and the untreated group (RLU_NT_) is ΔRLU_basal_, which reflects the P-gp ATPase activity:

RLU_Na3VO4_ − RLU_NT_ = ΔRLU_basal_

The D-value of the average luminescence intensity for the Na_3_VO_4_ group (RLU_Na3VO4_) and the test compound group (RLU_TC_) is ΔRLU_TC_, and ΔRLU_TC_ indicates the P-gp ATPase activity in the presence of a test compound.

RLU_Na3VO4_ – RLU_TC_ = ΔRLU_TC_

### 4.5. Membrane Fluidity 

The steady-state fluorescence polarization and fluorescence anisotropy were monitored as previously described [41]. The lipid-soluble fluorescent probe 1,6-diphenyl-1,3,5-hexatriene (DPH) was used. MDCK-MDR1 cells cultured for 4–5 days were washed twice to remove impurities. MDCK-MDR1 cells were incubated with different imperatorin concentrations (dissolved in 0.1% DMSO) or positive control drugs (cholesterol and benzyl alcohol) at 37 °C for 120 min. The culture liquid was discarded, and cells were washed with PBS 2–3 times. Cells were digested with trypsin-0.25% EDTA at room temperature and centrifuged at 1000 r·min^−1^ for 5 min. Cell cultures were discarded and resuspended in HBSS (pH 7.4). Cell numbers were quantified in a cell counter, and cell suspensions were diluted to 2 × 10^5^ cells·mL^−1^. The molecular probe DPH (100 µmol·L^−1^) was prepared in tetrahydrofuran, and light was avoided. The DPH solution (25 µL) was added to a 2.5-mL cell culture suspension, which was then mixed and incubated at 37 °C for 30 min. Measurements were taken using a PELS-55 fluorescence/phosphorescence/luminescence spectrophotometer (Perkin Elmer, Waltham, MA, USA) equipped with a polarizing filter. The excitation and emission wavelengths were 360 and 430 nm, respectively. The fluorescence anisotropy (r) of DPH and imperatorin–DPH embedded in the cell membrane was calculated using the following equation:

(1) $$r=\frac{I_{vv}-G•I_{vh}}{I_{vv}+G•2I_{vh}}$$

where *I_vv_* and *I_vh_* represent the perpendicular and parallel fluorescence intensities, respectively. *G* is the correction factor.

### 4.6. Rhodamine 123 Accumulation

Cells were inoculated in 24-well culture plates and cultured for 15 days. Different concentrations of imperatorin were diluted in HBSS. Cultures were digested into single cell suspensions, and the precipitates were collected via centrifugation (1500 rpm, 5 min). Cell suspensions (1 × 10^6^ cells/mL) were prepared in culture media, and 1-mL cell suspensions were inoculated into 2 mL Eppendorf tubes. Different drug concentrations were added in each group and incubated with cell monolayers at 37 °C for 30 min. Rh-123 (5.0 μM) was added to each group. Cold HBSS was used to wash cells four times to terminate P-gp-mediated transport after a 30 min incubation at 37 °C, and fluorescent dye not adsorbed to the outside of cells was discarded. Cells were extracted for 10 min with butanol, and Rh-123 fluorescence intensity was determined using a F-7000 fluorescence spectrophotometer (Ex = 515 nm; Em = 532 nm, Hitachi, Tokyo, Japan).

### 4.7. Effect of Imperatorin on P-gp Expression (Western Blotting) 

MDCK-MDR1 cells were cultured on 12-well plates at a density of 1 × 10^5^ cells/cm^2^ and grown for at least 4–5 days. The cells were incubated with different concentrations of imperatorin for 4 h, and cell monolayers were collected and washed 3 times with PBS. Total protein was extracted, and P-gp expression was assessed by western blotting (same method as Section 4.2.3).

### 4.8. qRT-PCR

MDCK-MDR1 cells were treated with different imperatorin concentrations (10 µg·mL^−1^, 5 µg·mL^−1^, and 1 µg·mL^−1^) for 6 h to determine whether the inhibition of P-gp-mediated drug transport was due to alterations of MDR1 mRNA. Total cellular RNA was extracted from the MDCK-MDR1 cells using the RNA easy Total RNA mini kit (Ambion, Beijing, China) according to the manufacturer’s instructions and reverse transcribed to single-stranded cDNA using a reverse transcription kit (Promega) according to the manufacturer’s protocol. PCR primer design and synthesis were performed by Shanghai Sangon Biotech (Shanghai, Beijing). MDR1 and hRpIPIv-F expression was quantified by PCR. The following primer pairs were used: human hRpIP1v-F forward, 5′-CCCTCATTCTGCACGACGAT; human hRpIP1v-F reverse, 5′-GGCTCAACATTTACACCGGC; human MDR1 forward, 5′-TGGGGCTGGACTTCCTCTCATGATGC, human MDR1 reverse, 5′-GCAGCAACCAGCACCCCAGCACCAAT. PCR amplification was performed using Power SYBR Green PCR Mix according to the manufacturer’s protocol (Life Technologies). Assays for each gene were performed in triplicate in 96-well optical plates using the ABI Prism 7500 sequence detection system (Applied Biosystems, Foster City, CA, USA).

### 4.9. Statistics

Results are expressed as the means ± SD of a minimum of four experiments. An analysis of variance (ANOVA) test was used to determine the statistical significance of differences between groups. The statistical significance of the differences in means was determined using Student’s t-test.

## Figures and Tables

**Table 1:** Detailed binding modes of ligands with P-gp and the LibDock Score.

Compounds	Type of Interaction	Key Amino Acids	LibDock Score
Imperatorin	Hydrogen Bond	leu971, phe332 and phe974	107
	Hydrophobic	ser975, phe71 and phe953	
Verapamil	r	gly222	132
	Hydrophobic	ala225	

**Table 2:** Effect of imperatorin on membrane fluidity (x ± sd, *n* = 4).

Group	*I_vv_*	*I_vh_*	Anisotropy
Control	566.923 ± 0.449	234.240 ± 0.081	0.103 ± 0.001
Imperatorin (10 µg/mL)	376.096 ± 6.845	151.398 ± 3.120	0.113 ± 0.002 *
Imperatorin (5 µg/mL)	429.017 ± 2.370	174.359 ± 1.395	0.109 ± 0.001 *
Imperatorin (1 µg/mL)	481.778 ± 8.710	197.003 ± 3.869	0.107 ± 0.002
Cholesterol (25 µm)	826.818 ± 6.157	288.311 ± 1.139	0.165 ± 0.001 **
Benzyl alcohol (30 mm)	540.317 ± 7.759	240.089 ± 3.142	0.077 ± 0.001 **

Compared with control group, * *p* < 0.05, ** *p* < 0.01.

**Table 3:** Effect of imperatorin on the accumulation of Rh-123 (x ± sd, *n* = 4).

Group	Dose (µg/mL)	Fluorescence Intensity
Control	-	35830 ± 7718
Rh-123	-	229466 ± 28022
Verapamil + Rh-123	10	428521 ± 34018 **
Imperatorin + Rh-123	1	258163 ± 16538
Imperatorin + Rh-123	5	294909 ± 35083 *
Imperatorin + Rh-123	10	339324 ± 8778 *

Compared with Rh-123, * *p* < 0.05, ** *p* < 0.01.

## Abbreviations

DPH	1,6-diphenyl-1,3,5-hexatriene
P-gp	P-glycoprotein
TCM	traditional Chinese medicine
FBS	fetal bovine serum
DMEM	Dulbecco’s modified Eagle’s medium
NEAA	non-essential amino acids
HBSS	Hank’s Balanced Salts Solution
Ver	Verapamil
